# Synthesis, Characterization and Cytotoxicity of Novel Multifunctional Fe_3_O_4_@SiO_2_@GdVO_4_:Dy^3+^ Core-Shell Nanocomposite as a Drug Carrier

**DOI:** 10.3390/ma9030149

**Published:** 2016-03-03

**Authors:** Bo Li, Huitao Fan, Qiang Zhao, Congcong Wang

**Affiliations:** College of Chemistry and Pharmaceutical Engineering, Nanyang Normal University, Nanyang 473061, China; libozzu0107@163.com (B.L.); zhaoqiang0522@126.com (Q.Z.); wccnanyang@163.com (C.W.)

**Keywords:** Fe_3_O_4_@SiO_2_@GdVO_4_:Dy^3+^, nanocomposites, core-shell structure, magnetic property, luminescence property

## Abstract

In this study, multifunctional Fe_3_O_4_@SiO_2_@GdVO_4_:Dy^3+^ nanocomposites were successfully synthesized via a two-step method. Their structure, luminescence and magnetic properties were characterized by X-ray diffraction (XRD), scanning electronic microscope (SEM), transmission electron microscopy (TEM), photoluminescence (PL) spectra and vibrating sample magnetometer (VSM). The results indicated that the as-prepared multifunctional composites displayed a well-defined core-shell structure. The composites show spherical morphology with a size distribution of around 360 nm. Additionally, the composites exhibit high saturation magnetization (20.40 emu/g) and excellent luminescence properties. The inner Fe_3_O_4_ cores and the outer GdVO_4_:Dy^3+^ layers endow the composites with good responsive magnetic properties and strong fluorescent properties, which endow the nanoparticles with great potential applications in drug delivery, magnetic resonance imaging, and marking and separating of cells *in vitro*.

## 1. Introduction

In recent years, controlled drug delivery systems for modern drug therapy have been attracting increasing attention because they exhibit low toxicity, a wide therapeutic window, and ideal drug efficacy as compared to conventional drug delivery systems [1,2]. The multifunctional nanocomposites combine with magnetic and luminescent properties in one entity, and they have attracted great attention in recent years owing to their potential application in the biotechnology and nanomedicine fields including magnetic resonance imaging (MRI), cell separation, drug delivery agents, cell separation, labeling, and optical probes [3,4,5]. In the choice of luminescent nanomaterials for labeling, targeting and imaging, lanthanide-doped nanomaterials possess many of advantages such as high fluorescence quantum yields, low toxicity, long lifetimes, and high stability in comparison to quantum dots and organic dyes [5,6,7,8].

So far, there have been some reports of constructing multifunctional nanomaterials that were made up of Fe_3_O_4_ and lanthanide-doped nanomaterials. In these reports [8,9,10,11,12], if the lanthanide-doped nanomaterials are chosen as cores, their luminescent intensity may be suppressed to some extent due to the coating of the outer layers. Meanwhile, if the lanthanide-doped nanomaterials are in direct contact with Fe_3_O_4_, their luminescence may be decreased as the direct contact can cause fluorescence-quenching [13,14,15]. Therefore, a SiO_2_ mid-layer between Fe_3_O_4_ and lanthanide-doped nanomaterials is needed.

To the best of our knowledge, there are no previous reports on the combination of magnetic properties with gadolinium vanadate nanophosphors. The previous investigation results indicated that nanosized GdVO_4_:Ln^3+^ phosphors have a significant application in a high definition flat display panels and potential applications in biology [16,17,18,19]. Compared with Ln^3+^-activated YVO_4_, GdVO_4_:Ln^3+^ exhibits highly efficient emitting phosphors, in which the energy transfers from the GdVO_4_ host to the incorporated Ln^3+^ ions through V^5+^–O^2−^ charge transfer (CT), yielding an efficient luminescence of Ln^3+^ activators.

Herein, we develop, for the first time, a novel and simple route to prepare Fe_3_O_4_@SiO_2_@GdVO_4_:Dy^3+^ core-shell microspheres with excellent magnetic and luminescence properties. The good aqueous colloidal stability, low toxicity and excellent self-heating efficacy make these novel magnetic, luminescent nanomaterials suitable for the hyperthermia treatment of cancer, and the luminescent entity helps us to identify the location of magnetic nanoparticles during *in vitro* cellular imaging [20,21,22,23,24].

## 2. Results and Discussion

**Figure 1:**
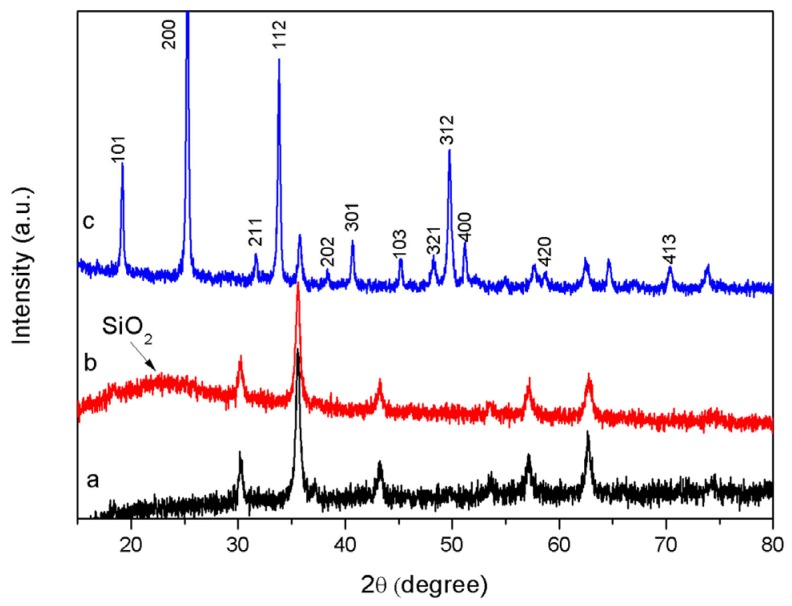
X-ray diffraction (XRD) patterns of pure Fe_3_O_4_ (**a**); Fe_3_O_4_@SiO_2_ (**b**) and Fe_3_O_4_@SiO_2_@GdVO_4_:Dy^3+^ (**c**). The diffraction peaks that are indexed in 1c correspond to GdVO_4_.

Figure 1 depicts the X-ray diffraction (XRD) patterns of as-synthesized Fe_3_O_4_, Fe_3_O_4_@SiO_2_ and Fe_3_O_4_@SiO_2_@GdVO_4_:Dy^3+^ nanoparticles. From Figure 1, we can find that there are characteristic diffraction peaks of Fe_3_O_4_, with a face-centered-cubic structure in all curves according to JCPDS card No. 65-3107. Besides the corresponding peaks of Fe_3_O_4_, SiO_2_ (JCPDS card No. 29-0085) and GdVO_4_ (JCPDS card No.86-0996) can be detected in Figure 1a–c, respectively. No peaks corresponding to impurities are detected, showing the adequate purity of the Fe_3_O_4_@SiO_2_@GdVO_4_:Dy^3+^ composites.

**Figure 2:**
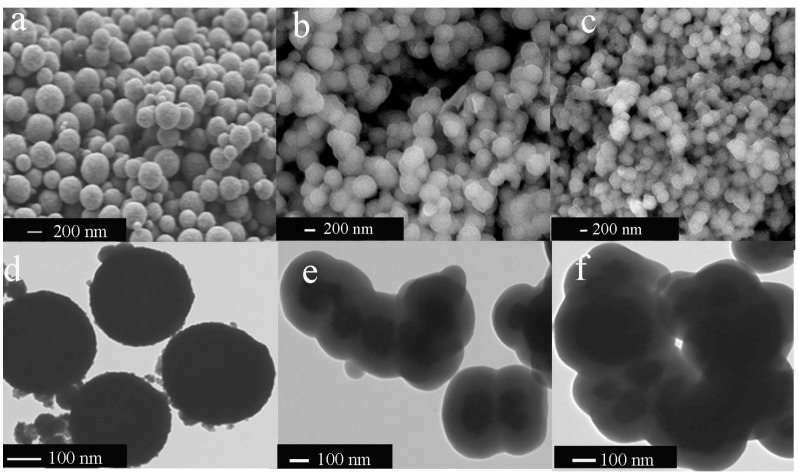
Scanning electronic microscope (SEM) images of Fe_3_O_4_ (**a**); Fe_3_O_4_@SiO_2_ (**b**); Fe_3_O_4_@SiO_2_@GdVO_4_:Dy^3+^ (**c**); and transmission electron microscopy (TEM) images of Fe_3_O_4_ (**d**); Fe_3_O_4_@SiO_2_ (**e**); Fe_3_O_4_@SiO_2_@GdVO_4_:Dy^3+^ (**f**).

The morphology and size details of the composites were characterized by SEM (scanning electronic microscope) and TEM (transmission electron microscopy) images. SEM investigations, as displayed in Figure 2a, reveal that the magnetic cores of Fe_3_O_4_ particles are of a rough appearance and have an average size of 290 (±20) nm. Once coated with one layer of silica, the composite microspheres are slightly larger in diameter and have a relatively smooth surface, with their size increased up to 320 (±30) nm, as shown in Figure 2b. The average size of the core-shell nanocomposites finally increased up to 360 (±25) nm, as illustrated in Figure 2c. The representative TEM images in Figure 2e,f indicate that the nanocomposites exhibit a core-shell structure.

**Figure 3:**
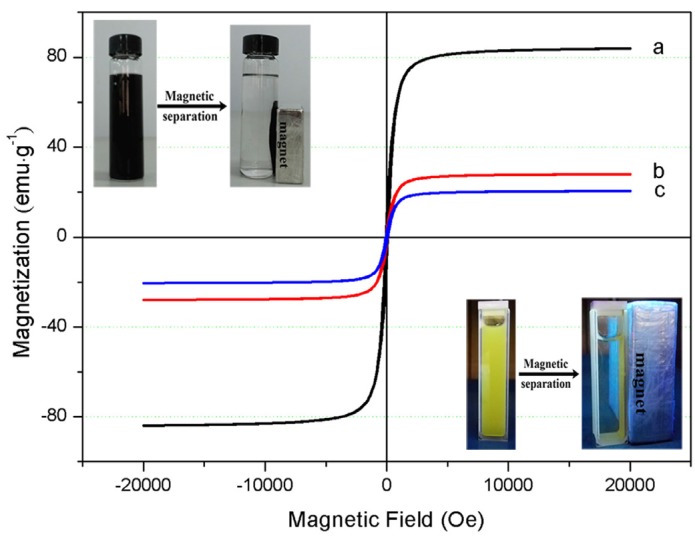
The magnetic hysteresis loops of pure Fe_3_O_4_ (**a**); Fe_3_O_4_@SiO_2_ (**b**); and Fe_3_O_4_@SiO_2_@GdVO_4_:Dy^3+^ (**c**).

To estimate the magnetic sensitivity, the room temperature magnetization hysteresis loops of the as-prepared cores and core-shell nanocomposites were collected and displayed in Figure 3. The magnetic hysteresis loops in Figure 3 indicate that they have saturation magnetizations of 83.9 emu/g (Fe_3_O_4_), 27.8 emu/g (Fe_3_O_4_@SiO_2_) and 20.4 emu/g (Fe_3_O_4_@SiO_2_@GdVO_4_:Dy^3+^) as well as negligible coercivity at room temperature, implying characteristics of their strong magnetism. The reduction of saturation magnetization could be attributed to the nonmagnetic shells (SiO_2_ and GdVO_4_:Dy^3+^). Our study revealed that, though the magnetism of the core-shell nanocomposites is less than that of the bare magnetic cores, it still possesses enough magnetic response for biomedical applications such as MRI, which is effectively magnetic separation.

**Figure 4:**
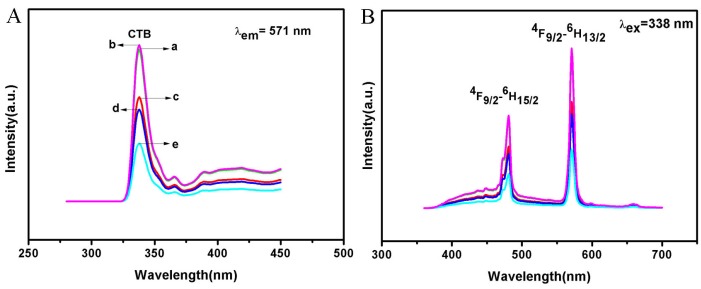
Excitation spectra (**A**) and emission spectra (**B**) of Fe_3_O_4_@SiO_2_@GdVO_4_:Dy^3+^ with different doped concentrations of Dy^3+^ (a: 0.5%, b: 1%, c: 2%, d: 3% and e: 4%).

The photoluminescence spectra of Fe_3_O_4_@SiO_2_@GdVO_4_:Dy^3+^ are shown in Figure 4. In the excitation spectra (Figure 4A), the excitation band at 300–350 nm monitored with a 571 nm emission of ^4^F_9/2_–^6^H1_3/2_ electronic transition of Dy^3+^ can be attributed to a charge transfer through the V–O bond overlay of the Dy–O charge transfer band. The emission spectra of GdVO_4_:Dy^3+^ are shown in Figure 4B. The main emission peaks at 481 nm and 571 nm are results of the ^4^F_9/2_–^6^H_15/2_ transition and ^4^F_9/2_–^6^H_13/2_ transition of Dy^3+^ ions. Moreover, Figure 4 shows the excitation spectra and emission spectra of Fe_3_O_4_@SiO_2_@GdVO_4_:Dy^3+^ composites with different doped concentrations of Dy^3+^ ions. It is shown that the optimum doped concentration of Dy^3+^ ions in the Fe_3_O_4_@SiO_2_@GdVO_4_:Dy^3+^ composites is 1 mol %.

**Figure 5:**
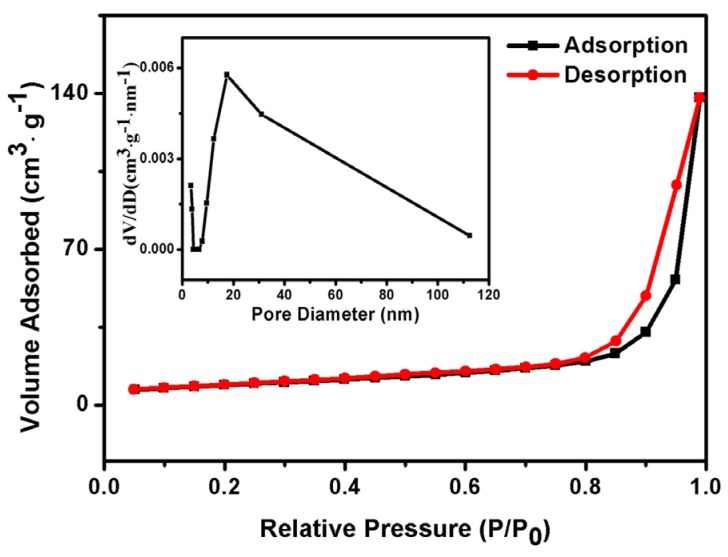
The N_2_ adsorption/desorption isotherms and pore size distribution (inset) of Fe_3_O_4_@SiO_2_@GdVO_4_:Dy^3+^.

To investigate the porous structure of the Fe_3_O_4_@SiO_2_@GdVO_4_:Dy^3+^ nanocomposites, the N_2_ adsorption-desorption isotherms were investigated and are shown in Figure 5. This isotherm profile can be categorized as type IV, with a small hysteresis loop observed at a relative pressure of 0.05–1.0, indicating the mesoporous features. The inset in Figure 5 is the pore size distribution. As calculated by the Brunauer-Emmett-Teller (BET) method, Fe_3_O_4_@SiO_2_@GdVO_4_:Dy^3+^ nanocomposites’ core-shell structure gives rise to a BET area of 30.21 m^2^·g^−1^, with a relatively high pore volume of 0.212 cm^3^·g^−1^, and the average pore diameter is 17.46 nm. The BET indicated the potential of such nanostructures for drug delivery applications.

**Figure 6:**
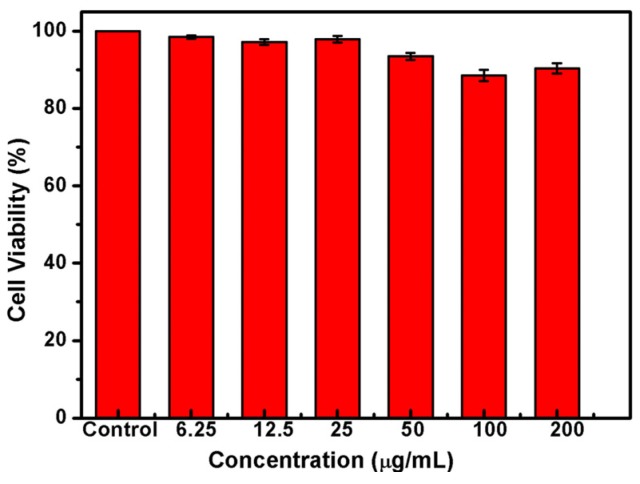
The viability histograms of HeLa cells incubated with different concentrations of Fe_3_O_4_@SiO_2_@GdVO_4_:Dy^3+^ nanoparticles for 24 h measured by a 3-(4,5-dimethylthiazol-2-y1)-2,5-diphenyltetrazolium bromide (MTT) assay.

To evaluate the cytotoxicity of the Fe_3_O_4_@SiO_2_@GdVO_4_:Dy^3+^ nanoparticles, *in vitro* cytotoxicity tests against HeLa cells were carried out. From the 3-[4,5-dimethylthiazol-2-y1]-2,5-diphenyltetrazolium bromide (MTT) viability histogram, shown in Figure 6, we can find that the Fe_3_O_4_@SiO_2_@GdVO_4_:Dy^3+^ causes insignificant damage to the HeLa cells when the sample concentration increases to 200 μg·mL^−1^ for 24 h, and the cell viability remains at 90.38% even at the highest concentration, which indicates that Fe_3_O_4_@SiO_2_@GdVO_4_:Dy^3+^ nanoparticles are biocompatible.

## 3. Materials and Methods

### 3.1. Materials

All reagents are of analytical reagent grade and used without further purification. Gd_2_O_3_ (99.9%) and Eu_2_O_3_ (99.9%) were purchased from Jinan Camolai Trading Company, Ferrous chloride hexahydrate (FeCl_3_·6H_2_O) (99%), tetraethyl orthosilicate (TEOS, 99.0%), sodium acetate (NaAc), Citrate acid monohydrate were purchased from Beijing Chemicals Corporation. Nitric acid, ethanol, ethylene glycol (EG), and ammonia aqueous (25%) were purchased from Tianjin Chemicals Corporation. Deionized water obtained from the Milli-Q system (Millipore, Bedford, MA, USA) was used in all experiments. The magnetic Fe_3_O_4_ nanoparticles were prepared using a modified solvothermal reaction.

### 3.2. Synthesis of Fe_3_O_4_

The magnetic Fe_3_O_4_ nanoparticles were prepared according to a previously reported synthetic process [19]. Typically, FeCl_3_·6H_2_O (1.3495 g) and NaAc (7.1926 g) were dissolved in EG solution (40 mL). Then PEG-10000 (1.0015 g) was added with vigorous stirring and the mixture was stirred for 30 min to form a homogeneous russet solution. The obtained solution was transferred to a Teflon-lined stainless-steel autoclave (50 mL capacity) and heated at 200 °C for 10 h. Subsequent cooling to room temperature yielded black magnetite particles, which were washed with ethanol and deionized water three times, respectively, and dried at 60 °C for 12 h.

### 3.3. Synthesis of Fe_3_O_4_@SiO_2_

Fe_3_O_4_@SiO_2_ nanoparticles were prepared according to the modified by the Stöber method. In brief, 1.0 g of Fe_3_O_4_ nanoparticles were homogeneously dispersed in a mixture of 160 mL of ethanol, 40 mL of deionized water, and 3.0 mL of 28 wt % concentrated ammonia aqueous solution, followed by the addition of 3.0 mL of tetraethyl orthosilicate (TEOS). After vigorous stirring at 40 °C for 6 h, the obtained Fe_3_O_4_@SiO_2_ microspheres were separated with a magnet and washed repeatedly with ethanol and deionized water to remove nonmagnetic by products.

### 3.4. Synthesis of Fe_3_O_4_@SiO_2_@GdVO_4_:Dy^3+^ Nanoparticles

**Scheme 1:**
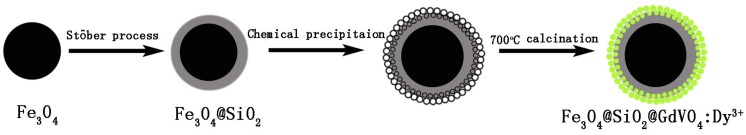
Illustration for the synthesis process of the spherical Fe_3_O_4_@SiO_2_@GdVO_4_:Dy^3+^ nanocomposite.

Functionalization of GdVO_4_:Dy^3+^ on the template Fe_3_O_4_@SiO_2_ was achieved according to the reported process with a doping concentration of Dy^3+^ of 0.5–4 mol % to Dy^3+^ in GdVO_4_:Dy^3+^. The typical procedure for synthesis is described as follows: stoichiometric amounts of Gd_2_O_3_, Dy_2_O_3_ and citric acid were dissolved in dilute nitric acid with heating followed by the addition of NH_4_VO_3_ in distilled water. Then PEG-10000 was added with a concentration of 0.05 g·mL^−1^. After stirring for 0.5 h, a homogenous sol was formed. Then the desired amount of Fe_3_O_4_@SiO_2_ nanoparticles was added into the gel, after further stirring for another 3 h, the resulting material was dried at 120 °C for 12 h to obtain the precursors. Then the precursors were calcined at 700 °C for another 4 h. The obtained nanoparticles were denoted as Fe_3_O_4_@SiO_2_@GdVO_4_:Dy^3+^ (Scheme 1).

### 3.5. Cytotoxicity Study of Fe_3_O_4_@SiO_2_@GdVO_4_:Dy^3+^ Nanoparticle

Cell viabilities of Fe_3_O_4_@SiO_2_@GdVO_4_:Dy^3+^ nanoparticles at different concentrations were tested by MTT assay on HeLa (human cervical cancer cells). In the experiment, the corresponding untreated cells were used as control. First, the HeLa cells were pre-incubated in a 96-well plate (about 3000 cells per well) for 24 h. Second, 2 mg of the Fe_3_O_4_@SiO_2_@GdVO_4_:Dy^3+^ nanoparticles were added into 10 mL of 0.01 M phosphate buffered saline (PBS, pH = 7.4) to form a stable orange solution. Third, the above solution at concentrations of 6.25, 12.5, 25, 50, 100 or 200 μg·mL^−1^ was added to the cells. Six parallel-group experiments were simultaneously conducted for each concentration. After 24 h, the viability of HeLa cells was examined by a MTT assay.

### 3.6. Characterization

The purities of all the nanoparticles were checked by X-ray diffraction (XRD) measurements at room temperature using Cu Kα radiation (Kα = 1.54059 Å). The morphology and microscope structure of all the nanocomposites were characterized by a scanning electronic microscope (SEM, NoVa™ Nano SEM 430, FEI Co., Ltd., Hillsboro, OR, USA) and transmission electron microscopy (TEM, JEOL JEM-2010F, JEOL Co., Ltd., Tokyo, Japan). The room temperature magnetic hysteresis (M-H) loops were measured using a superconducting quantum interference device vibrating sample magnetometry (SQUID-VSM, Quantum Design Co., Ltd., San Diego, CA, USA). Luminescence spectra were recorded on a FluoroMax-4 spectrophotometer (HORIBA Jobin Yvon Co., Ltd., Paris, France). The specific surface area was determined by the Brunauer-Emmett-Teller (BET) method. The HeLa cells were assayed for viability by using a microplate reader (Bio-Rad 680, Bio-Rad Co., Ltd., Hercules, CA, USA).

## 4. Conclusions

In summary, we report a novel magnetic/luminescence multifunctional nanocomposite, Fe_3_O_4_@SiO_2_@GdVO_4_:Dy^3+^, with a core-shell structure from a combination of hydrothermal reaction and the sol-gel process. The as-prepared nanocomposites, combining the merits of the good magnetic response of the assembled Fe_3_O_4_@SiO_2_ microspheres and the fluorescence property of GdVO_4_:Dy^3+^, displayed high surface area and biocompatibility. Therefore, our study may provide new insight and useful information for the design of diverse, functional nanocomposites as drug carriers.
